# Unexplained infertility categorization based on female laparoscopy and total motile sperm count, and its impact on cumulative live‐births after one in‐vitro fertilization cycle. A retrospective cohort study involving 721 cycles

**DOI:** 10.1002/rmb2.12368

**Published:** 2021-02-01

**Authors:** Ruma Satwik, Mohinder Kochhar

**Affiliations:** ^1^ Centre of IVF and Human Reproduction Institute of Obstetrics and Gynaecology Sir Ganga Ram Hospital New Delhi India

**Keywords:** female unexplained infertility, in‐vitro fertilization, laparoscopy, live‐births, male unexplained infertility

## Abstract

**Purpose:**

To determine how subcategorizing unexplained infertility based on female laparoscopy and total‐motile‐sperm‐count assessment would impact cumulative live‐births after one in‐vitro fertilization (IVF) cycle.

**Methods:**

Seven hundred twenty one IVF cycles from Jan 2014‐April 2019 performed at a single‐center were retrospectively analyzed. Couples with unexplained infertility having normal uterine and endometrial morphology were subcategorized into three cohorts, UI (1): those with no tuboperitoneal pathology on laparoscopy and total‐motile‐sperm‐count (TMSC) ≧20 million: n = 103; UI (2): tuboperitoneal pathology on laparoscopy or TMSC <20 million, n = 86; and UI(3): tuboperitoneal status not known: n = 114. Controls were severe male factor, bilateral tubal block, and grade 3/4 endometriosis: n = 418. Primary Outcome was cumulative‐live‐birth‐per‐initiated‐IVF cycle (CLBR). Odds ratio for live‐births were adjusted for confounding factors.

**Results:**

The CLBR in UI1 cohort was significantly lower than controls (29.1% vs 39; OR = 0.62; 95%CI = 0.39‐0.98; *P* = .04); but similar in UI2 and UI3 vs. controls. (37.2% vs 39.95%; OR = 0.89, 95%CI = 0.55‐1.44; *P* = .89) and (38.6% vs 39.95%; OR = 0.98, 95%CI = 0.64‐1.55; *P* = .98). After adjusting for age, infertility duration, past live‐births, and AMH, the adjusted odds for CLBR in UI1 was 0.48 (95%CI = 0.28‐0.82; *P* = .007).

**Conclusions:**

Unexplained infertility when defined after a normal laparoscopy and TMSC significantly lowered cumulative‐live‐births‐per‐initiated‐IVF cycle when compared with traditional diagnosis of tubal, endometriosis, or male factor infertility. In UI subcategory with abnormal laparoscopy or TMSC, CLBR remained unaffected. This information could be useful for counseling couples prior to IVF. Large‐scale prospective studies are needed to confirm this observation.

## Graphical abstract

This retrospective analysis of 721 cycles segregates unexplained infertility into a subcategory with normal female laparoscopy and total motile sperm count and finds that adjusted odds for live‐births is significantly lowered in this sub‐category vis‐a‐vis a known cause infertility after IVF treatment. The analysis makes adjustments for female age, infertility duration, past live‐births, past treatments, and AMH.

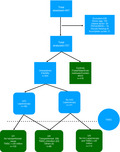

## 1 INTRODUCTION

Unexplained infertility (UI) is a diagnosis of exclusion made after standard infertility investigations involving tests of ovulation, tubal patency, and standard semen analysis have failed to reveal an underlying absolute cause as a barrier in causing natural conception.^1^

Existing literature is ambiguous about whether the diagnosis of UI has a better prognosis for live‐births in IVF when compared with the diagnoses of endometriosis, tubal or male factor infertility. In a retrospective analysis of 121 744 women undergoing their first cycle of autologous IVF between 2000‐2007, the authors found that UI had a better prognosis for live births vis‐à‐vis tubal or male factor infertility.^2^ However, in another cohort study involving 9915 women who underwent IVF/ICSI treatment from 2008 to 2012, the predictors found to be significantly associated with reduced chances of IVF/ICSI success among others were tubal factor and unexplained infertility.^3^ In yet another retrospective analysis of data collected from a randomized controlled trial involving 738 women, the odds of live‐birth following one‐cycle IVF were significantly lowered in the presence of tubal and male factor infertility when compared with UI (OR: 0.57; 95%CI: 0.38‐0.84 and 0.42, 95% CI 0.22‐0.79).^4^

This ambiguity in live‐birth prognosis could be because UI cannot be considered a monolith etiologically. It is a heterogenous condition encompassing subtle abnormalities in one or more of the following units: the fallopian tube, oocyte, sperm, or the endometrium.^5^ Specifically, the tubal defects may include, peritubal adhesions, fimbrial phimosis, or unilateral tubal block^6^; peritoneal defects may include minimal, mild, or moderate endometriosis; the sperm defects may include but not limited to, sperm concentration, motility or morphology abnormalities, defects in calcium oscillations leading to poor oocyte activation, etc^7^; oocyte defects may exist at follicular, cellular or molecular level encompassing genuine empty follicle syndrome, and oocyte nuclear or cytoplasmic defects leading to poor embryonic development^8^, ^9^;and finally, uterine defects, encompassing morphological or molecular endometrial abnormalities, in each case leading to poor implantation. These defects are subtle enough to be missed by a standard infertility work‐up.

Laparoscopy has been used to reasonably rule out tubal and peritoneal factors associated with unexplained infertility that cannot be diagnosed with tests such as HSG alone.^10^, ^11^, ^12^ A stricter criteria for assessing semen like total motile sperm count (TMSC) can rule out a sperm related factor, better than WHO 2010 lower reference limits, as a barrier in causing natural conception.^13^, ^14^, ^15^ It is known that IVF‐ICSI has the potential to overcome infertility due to failure of gamete migration to the site of fertilization or due to failure of sperm penetration into the oocyte. It is unlikely, however, to overcome infertility due to defects in oocyte, sperm or endometrium responsible for molecular events occurring post sperm entry into the oocyte such as oocyte activation,^16^ sperm decondensation, pronuclear alignment and fusion, cellular cleavage, embryonic genomic activation, blastulation, or implantation.^7^, ^17^ The absence of conditions potentially treatable by IVF‐ICSI (tubal, endometriosis, oligo‐astheno‐teratozoospermia) increases the chance of existence of molecular defects either in the gametes or endometrium. It is, therefore, hypothesized that in couples with a subcategory of UI who have normal female laparoscopy and TMSC, the live‐birth outcomes after IVF would be poorer when compared with couples undergoing IVF for potentially treatable factors like endometriosis, tubal or male factor infertility.

## 2 METHODS

The study was approved by the institutional ethics committee vide registration number EC/01/19/1465. Data of all couples undergoing IVF cycles performed between Jan 2014‐April 2019 at a tertiary care‐multiple‐provider set up was collected. The diagnostic categories of male, tubal, endometriosis, and unexplained infertility were included in the study. Exclusions were donor‐recipient cycles, preimplantation genetic testing, presence of gross uterine factor (diffuse adenomyosis, grade 2/3 Asherman's syndrome, uncorrected FIGO type 0, 1 or 2 fibroids, mid‐cycle endometrial thickness <6 mm) and incomplete cycles; defined when embryos from one oocyte aspiration cycle remained unutilized, in the absence of a live‐birth or ongoing pregnancy.

**FIGURE 1:**
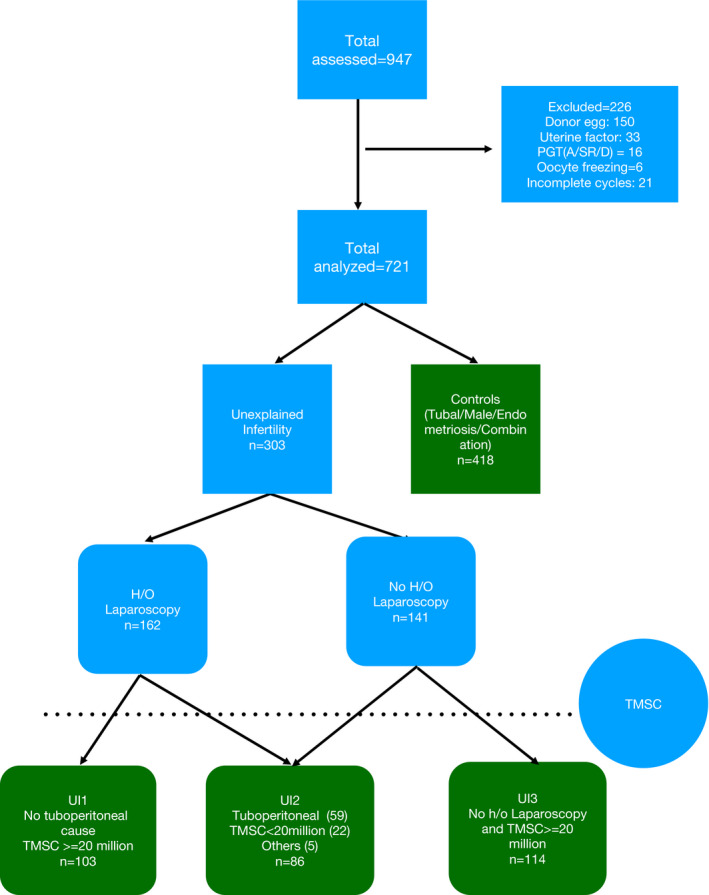
Flowchart showing cohort and control selection. "Others" indicate couples with non‐consummation and hypogonadotropic hypogonadism with normal semen analysis

The diagnostic categories of male, tubal, and endometriosis were regrouped into the control cohort. The diagnostic category of UI was subcategorized into three cohorts based on previous laparoscopy and TMSC findings as shown in Figure 1. Basically, previous laparoscopy findings were analyzed to define presence or absence of tuboperitoneal disease. Laparoscopic findings of unilateral tubal block, fimbrial phimosis, peri‐adnexal disease, pelvic adhesions, and grade 1/2 endometriosis constituted “tuboperitoneal disease.” The absence of above constituted “no tuboperitoneal disease.” In the absence of a prior laparoscopy, the status of tuboperitoneal disease was deemed “indeterminate.”

Semen reports of UI cycles were analyzed next. At least two semen reports (WHO 2010 standard) were available for all couples; one done prior to IVF initiation and one on IVF day. The poorer report was considered to calculate TMSC using the formula: total volume X sperm concentration X percentage of progressively motile sperm. TMSC of ≧20 million was deemed non‐sperm factor UI, and TMSC <20 million was labeled as mild‐sperm‐factor UI.

Hamilton et al (2015) have argued and shown that TMSC is a better parameter than WHO 2010 criteria to assess discriminating potential of sperm for natural pregnancy as it takes the absolute value of three semen parameters into consideration simultaneously.^13^ In addition, in 518 couples undergoing their first ICSI cycles, TMSC was shown to have a better predictive value over WHO lower reference limits for fertilization, blastulation, and miscarriage rates, and values more than 20 million/mL were suggestive of normal sperm function.^14^

Unexplained infertility cycles were finally subcategorized into three cohorts namely UI1: having non‐tuboperitoneal and non‐sperm factor unexplained infertility; UI2: having mild tuboperitoneal and/or mild‐sperm‐factor unexplained infertility; and UI3: having indeterminate factor, labeled so because in the absence of laparoscopy, a tuboperitoneal factor could not be ruled out. The distribution of different sub etiologies within the three cohorts of UI is shown in Table 1.

**TABLE 1:** Subcategorization of unexplained infertility and description of the four cohorts

Unexplained infertility cohorts	Description	Criteria	Total 721 n(%)
UI (1)	Non‐tuboperitoneal Non‐sperm factor UI	TMSC≧20million	103
		On laparoscopy:	
		No endometriosis	
		No pelvic adhesions	
		No tubal anatomical defect	
		B/L tubal patency	
UI (2)	Mild Tuboperitoneal or Mild‐sperm‐factor UI	Total	86
		Total motile sperm count < 20 million	22
		Unilateral Tubal block	17
		Previous ectopic with bilateral tubal patency	2
		Fimbrial phimosis on laparoscopy	12
		Peritubal adhesions altering tubo‐ovarian relationship on laparoscopy	12
		Minimal to mild endometriosis on laparoscopy defined as per AFS 1985 criteria	11
		Combinations and others	10
UI (3)	Indeterminate	Laparoscopy not done	114
		Tubal patency on HSG	
		TMC≧20 million	
Controls	B/L tubal block/Severe male/severe endometriosis	Total	418

### 2.1 Laparoscopy and in‐vitro fertilization

At the center of study, laparoscopy is not the first investigative modality to assess fallopian tubal patency. It is usually offered to women with at least three failed IUIs, UI with prior pelvic surgery, long‐duration UI usually exceeding two years, and positive pelvic findings on ultrasound or hysterosalpingography. Operative measures are undertaken during laparoscopy to address peri‐adnexal adhesions, cornual block, and endometriosis. Infertility state continuing after a period of one year with active attempts at pregnancy is considered failed laparoscopy, and IVF is offered subsequently.

In women with advanced age, long infertility durations, or with low AMH, an early IVF is offered and sometimes as the first treatment modality.

### 2.2 In‐vitro fertilization protocol

The protocol for IVF and transfer has been defined previously.^18^ In brief, all women underwent IVF in agonist or antagonist regimes with gonadotropin doses determined based on the woman's age, Body Mass Index (BMI), AMH, and previous response to ovarian stimulation. Fertilization of oocytes was achieved using conventional insemination or intracytoplasmic sperm injection. Embryos resulting from one cycle were transferred on day 2/3/5 in either fresh or subsequent frozen cycle till a live‐birth or ongoing pregnancy was achieved or embryos from one cycle were exhausted. Choice of elective freezing was made based on hCG day estradiol (>3000 ng/mL) endometrial thickness and pattern, appearance of symptoms of ovarian hyper stimulation syndrome, fever on the day of transfer, etc The frozen thawed embryo transfer cycle was/were undertaken in the subsequent months in hormonally prepared cycles for oligomenorrhoic women and natural cycles for eumenorrhoic women. Luteal phase support consisted of only vaginal micronized progesterone 400 mg twice daily in all cycles except where uterus had been hormonally prepared, in which case luteal support was given with alternating injectable 100 mg and vaginal progesterone 800 mg per day.

Demographic characteristics like age, infertility duration, past pregnancies, past infertility treatments, serum anti mullerian hormone(AMH) (ng/mL), and body mass index (BMI) were compared between the four cohorts. AMH was measured using Beckman Coulter Gen II assay till September 2016 and Access chemiluminescence assay subsequently. Values were reported in ng/ml. Where reported as pmol/L, (prior to September 2016) the values were converted into ng/mL using a divisor of 7.18.

### 2.3 Outcomes

Primary outcome variable was live‐birth resulting from the transfer of embryos generated per oocyte aspiration cycle. Live‐births were defined as all births at or beyond 26 weeks of gestation with survival for at least a day as per the hospital's neonatal intensive care guideline on the age of viability and active neonatal resuscitation policy. Crude and adjusted odds ratio for live‐births were calculated for all UI cohorts against controls.

Other outcomes assessed were total retrieved oocytes, mean total utilizable embryos, total‐eggs‐to‐utilisable‐embryo‐ratio, total‐eggs‐to‐live‐birth‐ratio, and mean‐transfers‐to‐one‐live‐birth. Total utilizable embryos were a sum of embryos/blastocysts transferred and embryos/blastocysts frozen in one oocyte retrieval cycle.

### 2.4 Statistical analysis

Data were expressed as mean ± 2 SD for gaussian variables and as median, interquartile range (IQR) for non‐gaussian variables. Independent samples t test and one‐way ANOVA were used to compare means between two groups and more than two groups, respectively, when the studied variable had a Gaussian distribution. Independent samples Kruskal‐Wallis test was used to compare medians for variables with non‐gaussian distribution. (Skewness quotient outside −1 to +1). Fischer's test and Chi‐square test were used to compare qualitative data when there were 2 and more than 2 groups, respectively. Binary logistic regression analysis was performed to calculate adjusted Odds ratio for live‐births adjusting for all confounders. A *P* value of <.05 was considered significant. All analyses were made on SPSS version 25.

## 3 RESULTS

### 3.1 Population characteristics

Nine hundred forty seven oocyte retrieval cycles were undertaken in the period of study. Two hundred twenty six were excluded on account of being incomplete (21), employing donor eggs (150) or preimplantation genetic testing (16), oocyte freezing (6), or having a visible uterine factor (33). Finally, 721 IVF cycles were analyzed. The overall mean female age in the included cycles was 32.56 ± 4.3 years, (range: 21‐44); median infertility duration was 6 years, (IQR: 4‐9); and previous live birth rate was 14%. 7.6% (n = 55) of cycles had no embryos for transfer, 74.3% (n = 536) had embryos for a single transfer only and 18.1% (n = 130) had embryos for ≥two transfer cycles. In 63.7% (n = 459) of cycles, fresh embryos were transferred and in 28.7%, (n = 207 cycles) only frozen embryos were transferred. A total of 788 transfers yielded 273 live‐births in this study giving a live‐birth rate of 34.6% per embryo transfer cycle and 37.9% per started cycle.

### 3.2 Comparison between the four cohorts

The number of cycles with diagnoses of UI1, UI2, UI3, and controls were 103, 86, 114, and 418, respectively. A comparison of pretreatment variables between the four cohorts is given in Table 2.

**TABLE 2:** Demographic and treatment parameters in the four cohorts

Parameter	UI (1) (103)	UI (2) (86)	UI (3) (114)	Controls (418)	*P* value
Age (y) (Mean ± SD)	33.59 ± 4.14	33.23 ± 4.11	33.2 ± 4.7	31.99 ± 4.25	.001
Infertility duration Median (IQR)	6 (4.5‐10)	6 (4‐9.1)	5 (3‐8)	6 (4‐9)	.033
Cycles with Previous live‐births n (%)	9 (8.7)	15 (17.44)	13 (11.2)	62 (14.8)	.00
BMI Kg/m^2^ (Mean ± SD)	26.29 ± 3.57	26.71 ± 5.06	25.5 ± 4.01	25.54 ± 4.13	.058
Cycles with previous ≥1 failed IUI. n (%)	87 (84.5)	52 (60.5)	87 (76.3)	104 (24.9)	<.001
Previous IUIs Median (Range)	3 (0‐12)	2 (0‐10)	2 (0‐19)	0 (0‐4)	<.001
Previous Failed IVFs n (%)	30 (29.1%)	22 (25.6%)	21 (18.4%)	101 (24.2%)	.31
AMH ng/mL Median (IQR)	3.9 (1.6‐6.3)	2.6 (1.2‐4.0)	2.2 (1.1‐4.6)	2.57 (1.4‐4.3)	.00
Total dose Median (IQR) IU	1575 (1200‐2250)	1775 (1400‐2212)	1650 (950‐2877)	1612 (1325‐1612)	.51
Estradiol Median (IQR) pg/mL	1900 (1082‐2837)	2004 (1165‐2993)	1746 (950‐2877)	1907 (932‐2355)	.074
Endometrial thickness (mm) Mean ± SD	8.96 ± 2.05	8.97 ± 2.04	8.6 ± 1.7	8.34 ± 1.95	.01

Note

Gaussian variables expressed as mean ± SD. Non‐Gaussian as median (interquartile range) or median (range). Categorical variables expressed as number and percentage

### 3.3 Outcomes

Despite a significantly higher numbers of oocytes retrieved in UI1 vs controls, (median 10 vs 8; *P* = .04) the median utilizable embryos remained at 2 for both these etiological categories. (*P* = .6). The live‐births per started cycle were significantly lower in UI1 compared with controls. (29.1% vs 39.95%, *P* = .042, two tailed and *P* = .023 one tailed). The eggs‐to‐utilizable‐embryo‐ratio, (4.5 vs 3.6; *P* = .029) eggs‐to‐live‐birth‐ratio, (39.4 vs 22.7; *P* = .007), and mean‐transfers‐to‐one‐livebirth (3.93 vs 0.2.7; *P* = .02) was poorer for UI1 compared with controls.

In UI2 and UI3 cohorts, live‐births were similar (37.2%, 38.6%, and 39.95%, *P* = .67 and .9) as were the median oocytes recovered (9, 8 vs 8; *P* = .06 and .54), the median utilizable embryos (2, 2 vs 2) the total‐eggs‐to‐live‐birth‐ratio (29.2, 25.9 vs 22.7; *P* = .34 and .53), and mean‐transfers‐to‐one‐live‐birth (3, 2.7 vs 2.7; *P* = .67 and .9) when compared with controls. (Table 3).

**TABLE 3:** IVF outcomes in the four cohorts

Outcomes	UI (1) (103)	UI (2) (86)	UI (3) (114)	Controls (n = 418)	*P* value 1 vs 4 2 vs 4 3 vs 4
Median oocytes retrieved (Range)	10 (0‐37)	9 (0‐41)	8 (0‐34)	8 (0‐37)	0.00 0.06 0.54
Median utilisable embryos (Range)	2 (0‐12)	2 (0‐8)	2 (0‐7)	2 (0‐8)	NS
Total oocytes retrieved (o)	1183	938	1140	3800	‐
Total utilizable embryos	264	205	266	1040	‐
Total transfer cycles	118	99	118	453	‐
Cumulative Live‐births (CLB)^a^ n (%)	30 (29.1%)	32 (37.21%)	44 (38.6%)	167 (39.95%)	0.04 0.63 0.9
Oocytes to embryo ratio^b^	4.5	4.6	4.3	3.6	0.02 0.01 0.03
Oocytes to live‐birth ratio^c^	39.4	29.2	25.9	22.7	0.007 0.34 0.53
Mean transfers to live‐birth ratio^d^	3.93	3	2.7	2.7	0.02 0.67 0.9

^a^ Cumulative live‐birth = Live‐birth resulting from all transfers from one initiated IVF cycle.

^b^ Oocytes to embryo ratio = Total oocytes needed to make one utilizable embryo, obtained by ratio of total retrieved oocytes to total utilizable embryos.

^c^ Oocytes to‐live‐birth‐ratio = mean eggs needed to produce one live‐birth.

^d^ Mean transfers to live birth‐ratio = Total transfers that produced one live‐birth

The crude odds ratio for live‐births against controls for the UI1 cohort was 0.62 (95%CI 0.39‐0.98, *P* = .043). The adjusted odds ratio for live‐births adjusting for confounding variables age, infertility duration, past live‐births, past treatments, and AMH was 0.48 (95% CI = 0.28‐0.82; *P* = .004). The crude and adjusted odds ratio for live‐births in the UI2 and UI3 subcategory against controls were not significant (Table 4).

**TABLE 4:** Crude and adjusted odds for live‐births with 95% Confidence Intervals

Confounding variable	Crude OR	95%CI	Adjusted odds	95%CI	*P* value
Age	0.95	0.93‐0.97	0.955	0.91‐0.99	.038
Infertility duration	0.83	0.76‐0.91	0.946	0.906‐0.99	.022
UI (1) vs controls	0.62	0.39‐0.98	0.51	0.3‐0.86	.012
UI (2) vs controls	0.89	0.55‐1.44	0.905	0.54‐1.5	.75
UI (3) vs controls	0.98	0.64‐1.50	0.85	0.53‐1.35	.54
Previous live‐births vs. none	1.02	0.67‐1.56	1.29	0.77‐2.18	.65
No IVF vs Previous IVFs	1.26	0.9‐1.8	1.21	0.83‐1.76	.33
AMH	1.44	1.25‐1.65	1.13	1.07‐1.19	.000

## 4 DISCUSSION

Unexplained Infertility (UI) encompasses various conditions arising due to defects in one or the other unit of reproduction and is considered a diagnosis of exclusion incumbent upon normalcy of semen analysis, tubal patency, and ovulation tests. The greater the armamentarium of investigations employed, the narrower the prevalence of UI is likely to be.^19^

In a study that employed routine laparoscopy in women with UI, (where prior tubal patency had been established on HSG), 25% additional cases of pelvic pathology could be discovered, encompassing severe tubal disease (4%), peri‐adnexal adhesions (8%), and Grade 1/2 endometriosis (13%).^6^ Our study showed additional tuboperitoneal pathology in 36% (59/162) of women diagnosed with UI who underwent laparoscopy.

On the question of whether uncovering these subtle pelvic pathologies made a difference in terms of IUI results, the same authors, (Tanahatoe et al^20^), could prove through a separate exquisitely designed RCT, that the impact of this laparoscopic detection and treatment of observed pelvic pathology was negligible in terms of IUI outcome, and hence a routine laparoscopy prior to IUI was not recommended.

Similarly, Siristatidis and Bhattacharya argued against instituting extensive investigations to unearth causes of UI like mild tubal disease, age related infertility, endometriosis, or immunological causes leading to infertility since they surmised that uncovering an exact diagnosis of UI does not change the management of these conditions and that the choice of treatment whether expectant, IUI, or IVF continues to be guided by the woman's age and infertility duration irrespective of the unearthed cause.^21^ Also, the guidelines for the diagnosis and management of UI formulated over the years based on the gradually accumulating evidence have reaffirmed the aforementioned standpoint.^22^, ^23^

While the need to institute extra investigations to uncover UI etiologies may not make pragmatic sense from the management standpoint, this information might make sense from a *prognostic standpoint* in IVF. While it is understood that a significant proportion of women with mild tuboperitoneal disease may conceive spontaneously or by IUI treatments, those that do not may be presumed to have an IVF‐amenable‐tuboperitoneal factor at a greater frequency than women who do not have mild tuboperitoneal disease. A previously done laparoscopy may help us in identifying such women.

So far, the prognosis for UI in IVF has been deemed either superior,^2^, ^4^ similar,^24^, ^25^ or inferior^3^, ^26^, ^27^ to those undergoing IVF for other indications. The ambiguity may be because of the heterogenous nature of UI. It is not known yet whether division of UI into more homogenous subcategories would make the prognosis for live‐births in IVF different across categories.

The present study divided UI into a non‐tuboperitoneal‐non‐sperm‐factor cohort and a mild‐tuboperitoneal/mild‐sperm‐factor cohort, after application of laparoscopy findings and total motile sperm count. In doing so, it attempted to address the hypothesis that IVF‐ICSI would be potentially less successful in the non‐tuboperitoneal‐non‐sperm‐factor cohort, where embryogenesis or implantation events downstream of sperm penetration into the oocyte were most likely to be affected.

This study showed that couples with UI defined after normal laparoscopy and TMSC of ≧20 million (representing non‐tuboperitoneal, non‐sperm factor infertility) had fewer live‐births per started cycle compared with couples undergoing IVF for a severe tuboperitoneal or severe sperm factor. (OR, 0.62; 95% CI = 0.32‐0.98, *P* = .04). The difference in live‐births remained significant, infact even more enhanced after adjusting for cofounding factors. (OR = 0.48; 95% CI = 0.28‐0.82), suggesting that if age, infertility duration, past live‐births, and AMH were equal in the two groups, the odds for a live‐birth after one cycle of IVF in couples with UI1 would be reduced to half when compared with couples with a known factor infertility.

Also it was seen that in the UI1 cohort, despite a mean higher number of retrieved eggs, the numbers of utilizable embryos were similar to controls. And despite a mean higher number of transfers in this cohort, the live‐births remained lower. Together, the above findings appear to suggest, that in unexplained infertility with normal laparoscopy and total motile sperm count, sub‐microscopic gamete, or endometrial defects predominate as etiologic factor and hence are potentially less amenable to IVF‐ICSI.

Endometrial implantation potential has been found to be lower in some cases of unexplained infertility due to chronic endometritis,^28^ alteration of vaginal flora, alteration in endometrial blood flow,^29^ or in the expression of proimplantation molecules.^30^ But uterine causes are a rare cause of infertility ^31^ and may not contribute to more than 10%‐15% of all cases of true unexplained infertility. ^7^

Since it was seen that true unexplained infertility relied on supernumerary embryos and multiple transfers to overcome infertility, it is presumed that the defect most likely lay at the embryonic level with some embryos having the potential and most not having the potential for live‐birth. Further it may be said that since AMH strongly correlates with retrieved oocyte numbers, which in turn correlates positively with supernumerary embryos, AMH might correlate better with live births in true unexplained infertility.

**FIGURE 2:**
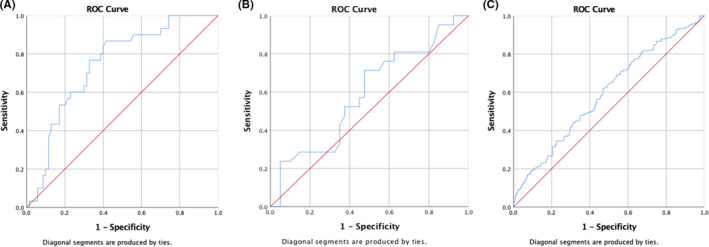
ROC curves depicting the correlation between AMH and cumulative live‐births in the UI1, UI2, and control cohorts

To check this hypothesis, a correlation of AMH value with cumulative live‐births in UI cohorts and controls was performed using ROC curves. (Figure 2) The analysis shows AMH to be a strong predictor of cumulative live‐births in UI (1) cohort representing true unexplained infertility; AUC = 0.75; 95% CI = 0.65‐0.85 but not so in UI(2), AUC = 0.59;95%CI = 0.44‐0.74 or controls; AUC = 0.59; 95% CI 0.54‐0.69. This could be a novel finding of our study that AMH has a strong positive correlation with cumulative live‐births in couples with true unexplained infertility but not in other etiological categories, but it requires further validation. Analysis of SART data so far finds an overall low predictive capability of AMH for live‐births in IVFs in unselected etiological categories. (AUC: 0.53 for FETs; and 0.63 for fresh cycles).^32^

### 4.1 Age, infertility duration and AMH

It is noted that female age is marginally higher by 1.2 to 1.5 years in UI (1, 2 and 3) versus controls. The higher age at IVF in UI could be a result of longer time spent in other infertility treatments like expectant management or IUI as can be seen from Table 2. Maheshwari, Hamilton, and Bhattacharya, (2008) have made the observation that UI is a diagnosis made more often in women ≥35 years than in women younger than 35 years.^33^ However, the outcomes in UI2 and UI3 categories remained similar to controls despite mean female age being higher in these cohorts than controls. Hence, it is suggested that the negative effect of UI1 on live‐births extends beyond age.

It is imperative to note that the median infertility duration of couples in all cohorts in this study was 6 years. (interquartile range 4‐9 years., mean: 6.95 ± 4.3). This is higher than the median infertility durations of 4 years (IQR 3‐6) stated in other studies that employed the HFEA database^2^, ^3^, ^27^; or the mean infertility duration of 3.9 ± 2.5 years in the study by Pettersson et al, (2010). The high median duration of infertility is in concordance with other studies done from the same geographic area.^34^, ^35^ Infertility duration is a strong negative determinant of live‐births in IVF. This factor has to be kept in mind while interpreting the results of this study.

It is also essential to note that median AMH is higher in UI1 cohort compared with controls despite median age being contrarily higher. The higher AMH in UI1 cohort could reflect a selection bias probably suggesting either an elimination of women with very low AMH or an inadvertent inclusion of more women with PCOS. Could a higher prevalence of PCOS in UI1 have negatively influenced outcomes?

To answer this question, an analysis of AMH distribution among the four cohorts was done (Table 5). A significantly higher percentage of women in UI1 had AMH >3.5 ng/mL than in UI2, UI3, or controls. (52.4% 29.1%, 35.1%, and 33.4%; *P* = .001). However, the low, average, and high AMH categories represented by 18.5%, 28.1%, and 52.4% of the UI1 cohort, accounted for 0%, 16.66%, and 83.33% of the total live‐births in that cohort. This suggested that a higher prevalence of AMH >3.5 in UI1, probably representing PCOS, did not negatively affect live‐births in this cohort. If at all, the results suggest, that if the UI1 cohort was matched with controls by AMH, the live‐birth rates in UI1 would have been even lower than that obtained in this study.

**TABLE 5:** The prevalence of low average and high AMH in the four cohorts and Live‐births stratified by AMH in the four cohorts

AMH ng/mL		UI (1)		UI (2)		UI (3)		Controls		*P* value
		Cycles	Live‐births	Cycles	Live‐births	Cycles	Live‐births	Cycles	Live‐births	
		n = 103	n = 30	n = 86	n = 32	n = 114	n = 44	n = 418	n = 167	
<1.2	Prevalence n'/n X 100	18.5	0	22.1	12.5	30.7	13.6	21.8	15	.001 for AMH prevalence across cohorts and .002 for live‐births stratified by AMH across cohorts
1.2‐3.5	Prevalencen'/nX100	29.1	16.7	47.6	43.7	33.3	38.6	44.8	45.5	
>3.5	Prevalencen'/nX100	52.4	83.3	29.1	43.8	35.1	47.7	33.4	39.5	

The findings that a rigorous categorization of UI might have implications for prognosis in IVF are novel. It attempts to glean information from already available data like previous laparoscopy and total motile sperm count to identify couples with poorer prognosis in IVF without additional cost to couples. This information when coupled with known predictors like age, infertility duration, past live‐births, and ovarian reserve parameters might add to the predictive value of existing models of live‐birth prediction in IVF.

This is a retrospective analysis and although attempts have been made to address biases, the study needs external validation through prospectively done studies within more strictly selected populations.

In conclusion, unexplained infertility, when defined after a normal laparoscopy and a normal total motile sperm count significantly lowers cumulative live‐births‐per‐started‐IVF cycle when compared with traditional diagnosis of tubal factor, endometriosis‐associated, or male factor infertility. The difference persists despite adjustments for age, previous live births, AMH, and infertility duration. In unexplained infertility subcategory with abnormal laparoscopy or TMSC, the live‐birth rates in IVF remain unaffected when compared with couples with traditional diagnosis of tubal factor, endometriosis‐associated, or male factor infertility. This information could be useful for counseling couples prior to IVF. Also, based on the mean embryo to live‐birth ratio and the mean transfer to live‐birth ratio, the couple with true unexplained infertility, especially those with long‐duration infertility, could be counseled that, they may need more total cycles to achieve a live‐birth than their other counterparts.

## DISCLOSURES

Conflict of interest statement: The authors declare that they have no conflict of interest.

Human rights statements and informed consent: This was a retrospective observational study. All data were anonymized and consent waiver sought from the ethics committee of the institution for this retrospective observational study. Approval was granted by the institutional ethics committee via registration number EC/01/19/1465.

Animal studies: This article does not contain any studies/intervention with human/animal subjects performed by any of the authors.

Approval by ethics committee: The study was conducted after the due formal approval of institutional ethics committee vide registration number EC/01/19/1465.

Clinical trial Registry: Not applicable. This was a retrospective observational study.
